# Artificial intelligence–based anatomical recognition improves surgeon decision-making during robotic gastrectomy

**DOI:** 10.1007/s10120-026-01756-5

**Published:** 2026-05-19

**Authors:** Kenichi Ishibayashi, Noriyuki Inaki, Jumpei Ikeda, Kenta Doden, Masashi Takeuchi, Kengo Hayashi, Ryota Matsui, Satoru Matsuda, Toshikatsu Tsuji, Jun Kinoshita, Hirofumi Kawakubo, Yuko Kitagawa

**Affiliations:** 1 Department of Gastrointestinal Surgery, Kanazawa University Hospital, 13-1 Takaramachi, Kanazawa, Ishikawa 920-8641 Japan; 2 Department of Surgery, Keio University School of Medicine, 35 Shinanomachi, Shinjuku-Ku, Tokyo 160-8582 Japan; 3 Direava, Inc, Tokyo, Japan

**Keywords:** Gastric cancer, Gastrectomy, Robotic surgery, Artificial intelligence, Deep learning

## Abstract

**Background:**

Artificial intelligence (AI)–based anatomical recognition has emerged to support intraoperative cognition; however, its clinical utility beyond education remains limited. This study aimed to develop an AI model for suprapancreatic lymph node dissection during robotic distal gastrectomy (RDG) and evaluate its utility for intraoperative decision-making by surgeons.

**Methods:**

We developed a deep learning model using 67 RDG videos (54 for training and 13 for testing) to recognize the pancreas, common hepatic artery (CHA), left gastric artery (LGA), and left gastric vein (LGV). Model performance was evaluated using Intersection over Union (IoU). Twenty surgeons participated in two experiments: experiment 1 assessed peritoneal incision line selection, rated by three experts on a 5-point scale, and experiment 2 assessed the time to CHA identification in a crossover design.

**Results:**

The IoU values for the pancreas, CHA, LGA, and LGV were 0.66, 0.28, 0.216, and 0.232, respectively. In mixed-effects models, experiment 1 showed that AI assistance reduced the proportion of unsafe peritoneal incision lines (scores 1–2) compared with no assistance (odds ratio, 0.25; 95% confidence interval [CI], 0.15 to 0.41; *P* < 0.001) and improved the mean expert score by 0.64 points (95% CI, 0.39 to 0.89; *P* < 0.001). In experiment 2, AI assistance reduced the time to CHA identification by 9.5 s (95% CI, 2.20 to 16.81; *P* = 0.0124).

**Conclusion:**

Our AI system successfully supported surgeons’ intraoperative decision-making by improving anatomical recognition and thus can potentially enhance surgical safety.

**Supplementary Information:**

The online version contains supplementary material available at 10.1007/s10120-026-01756-5.

## Introduction

Gastric cancer is one of the most common malignant tumors and the fifth leading cause of cancer-related deaths worldwide [1]. Despite improvements in non-surgical treatments such as immune checkpoint inhibitors, the most promising curative therapy for gastric cancer to achieve long-term survival is gastrectomy [2, 3]. Over the past few decades, the surgical approach has evolved from open surgery to laparoscopic gastrectomy and, more recently, robotic gastrectomy (RG) [4]. By providing articulation, tremor filtration, and stable three-dimensional visualization, RG overcomes the inherent limitations of conventional laparoscopy and enables a more precise dissection [5–8]. However, despite these technological advancements, postoperative complications, including pancreatic fistula, abdominal abscess, and bleeding, remain an important concern. Major complications (grade II or higher) still occur in 7.7–8.8% of cases after gastrectomy [7–10]. These adverse events prolong hospitalization and increase healthcare costs while negatively affecting long-term oncologic outcomes [11–13]. Therefore, further improvements in surgical safety are required. Surgical outcomes depend not only on technical skills but also on intraoperative cognition and decision-making. Notably, more than half of the intraoperative adverse events have been attributed to human performance deficiencies, highlighting the pressing need for innovative technologies that support surgeons’ intraoperative cognitive abilities to enhance safety and precision in gastric cancer surgery [14].

Recently, artificial intelligence (AI)–based anatomical recognition has emerged as a tool for supporting intraoperative surgical cognition [15, 16]. Many studies have demonstrated the feasibility of deep learning models for identifying major organs, critical landmarks, and surgical phases across various procedures, including gastrectomy [17, 18]. However, these systems are primarily restricted to student education [19]. For instance, Nakao et al. recently demonstrated that AI-based visualization significantly improved medical students’ sensitivity in identifying the pancreas during RG compared with conventional instruction [20]. As such, the clinical utility of directly supporting surgeons is yet to be fully demonstrated. To serve as a clinically useful system, AI models must enhance recognition accuracy and enable surgeons to identify *structures* at an earlier surgical phase. In contrast to these predominantly educational systems, we previously developed an AI-based anatomical recognition model for robotic distal gastrectomy (RDG) and demonstrated its ability to enhance the recognition accuracy of surgeons in the pancreas and transverse mesocolon [21]. Therefore, the next phase was to verify whether the AI model could support anatomical recognition and directly improve the quality of the intraoperative decision-making process.

In this pre-clinical IDEAL (Idea, Development, Exploration, Assessment, Long-term study) Stage 0 study, we aimed to develop an AI-based anatomical recognition model for suprapancreatic lymph node dissection during RDG and examine whether it supports surgeons’ anatomical recognition and intraoperative decision-making. We assessed its clinical value by evaluating its effects on peritoneal incision line selection and the time required to identify the common hepatic artery (CHA) to explore the role of the AI model as a decision support system in RDG.

## Methods

### Dataset

We included 67 patients with gastric cancer who underwent RDG with D1 + or D2 lymphadenectomy at Kanazawa University Hospital and Keio University Hospital. This study was approved by the ethics committees of both institutions (approval numbers: Kanazawa University Hospital, 814725-1 and Keio University Hospital, 20231207). All procedures were performed by board-certified surgeons. Written informed consent was obtained from all participants before their inclusion in the study.

### Surgical procedure for suprapancreatic lymph node dissection in RDG

The da Vinci Xi system (Intuitive Surgical, Sunnyvale, CA, USA) and Hinotori surgical system (Medicaroid, Kobe, Japan) were used for RDG. Patients were placed in the supine or open-leg position. Accordingly, four ports designated for the robots and another port designated for the assistant were established. In all procedures, suprapancreatic lymph node dissection was initiated with a peritoneal incision along the pancreas. The procedure continued with dissection along the outermost layer of the CHA, left gastric artery (LGA), and left gastric vein (LGV), followed by the division of the roots of the LGA. Finally, the gastric body was transected using a linear stapler.

### Development of the AI algorithm

For the AI model development, 67 surgical videos from two institutions were used, of which 54 were used as training data and 13 were used as test data. From the 54 videos, we extracted scenes from the beginning to the end of the suprapancreatic lymph node dissection, randomly selecting 30 images as training data. We annotated the contours of vital organs, including the pancreas, CHA, LGA, and LGV, for AI model training. A board-certified surgeon in gastroenterology annotated each organ, and an expert surgeon confirmed their quality by making the final annotation. To mitigate the risk of overfitting associated with the limited dataset size and ensure robust generalization, we used a comprehensive data augmentation pipeline during training. Specifically, our strategy incorporated a diverse combination of spatial transformations, photometric distortions, and domain-specific occlusion techniques*.* In all modeling procedures, a script written in Python 3.7 (Python Software Foundation, Beaverton, OR, USA; https://www.python.org/downloads/release/python-370/) was used. Furthermore, a computer equipped with an NVIDIA GeForce RTX 3090 graphics-processing unit (NVIDIA, Santa Clara, CA, USA) and an Intel^®^ Core™ (Intel, Santa Clara, CA, USA) central processing unit i9-10900X @ 3.70 GHz with 128 GB of random-access memory was used for model training with support from Direava, Inc.

### Evaluation of AI performance

Thirteen surgical videos from two hospitals that were not included in the training dataset were used to evaluate the AI model. Using these videos, we randomly selected 30 frames from the suprapancreatic lymph node dissection scenes. Frames unsuitable for annotation, including distant views from the camera and blurred screens caused by surgical smoke, bleeding, or stains, were excluded. The performance of the algorithmic segmentation methods was measured using the Intersection over Union (IoU), which is a metric used to evaluate how well a deep learning network’s predicted segmentation mask aligns with ground truth data annotated by an expert surgeon. To calculate the IoU, we divided the overlap between the predicted segmentation mask and the ground truth by the union of these two sets. In evaluating model performance, an IoU of 1 indicated perfect segmentation and 0 indicated no overlap between the model-segmented and manually annotated regions.

### Evaluation of the clinical utility of AI by surgeons

**Fig. 1:**
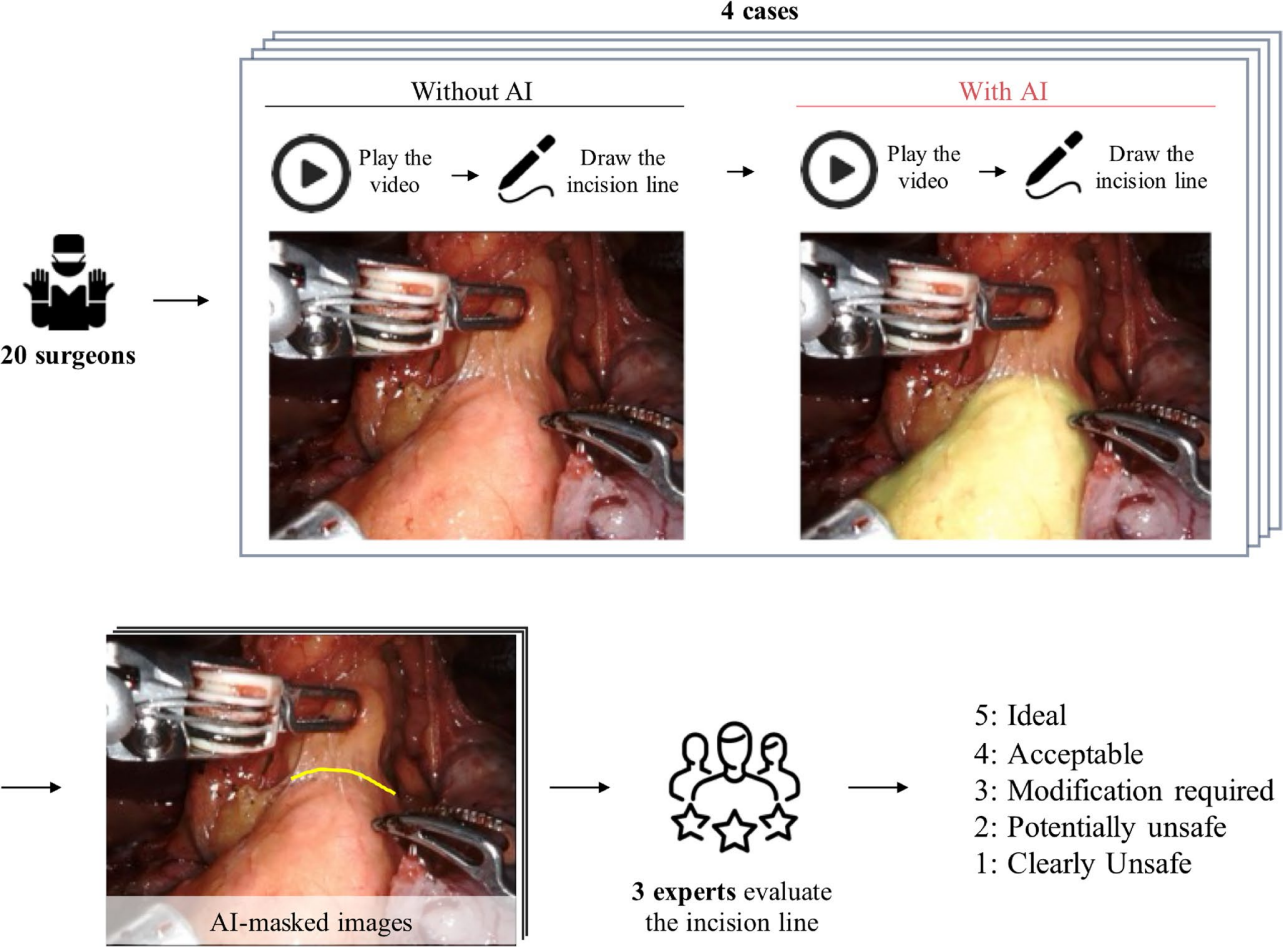
Workflow of experiment 1 assessing peritoneal incision line selection with and without AI assistance.

**Fig. 2:**
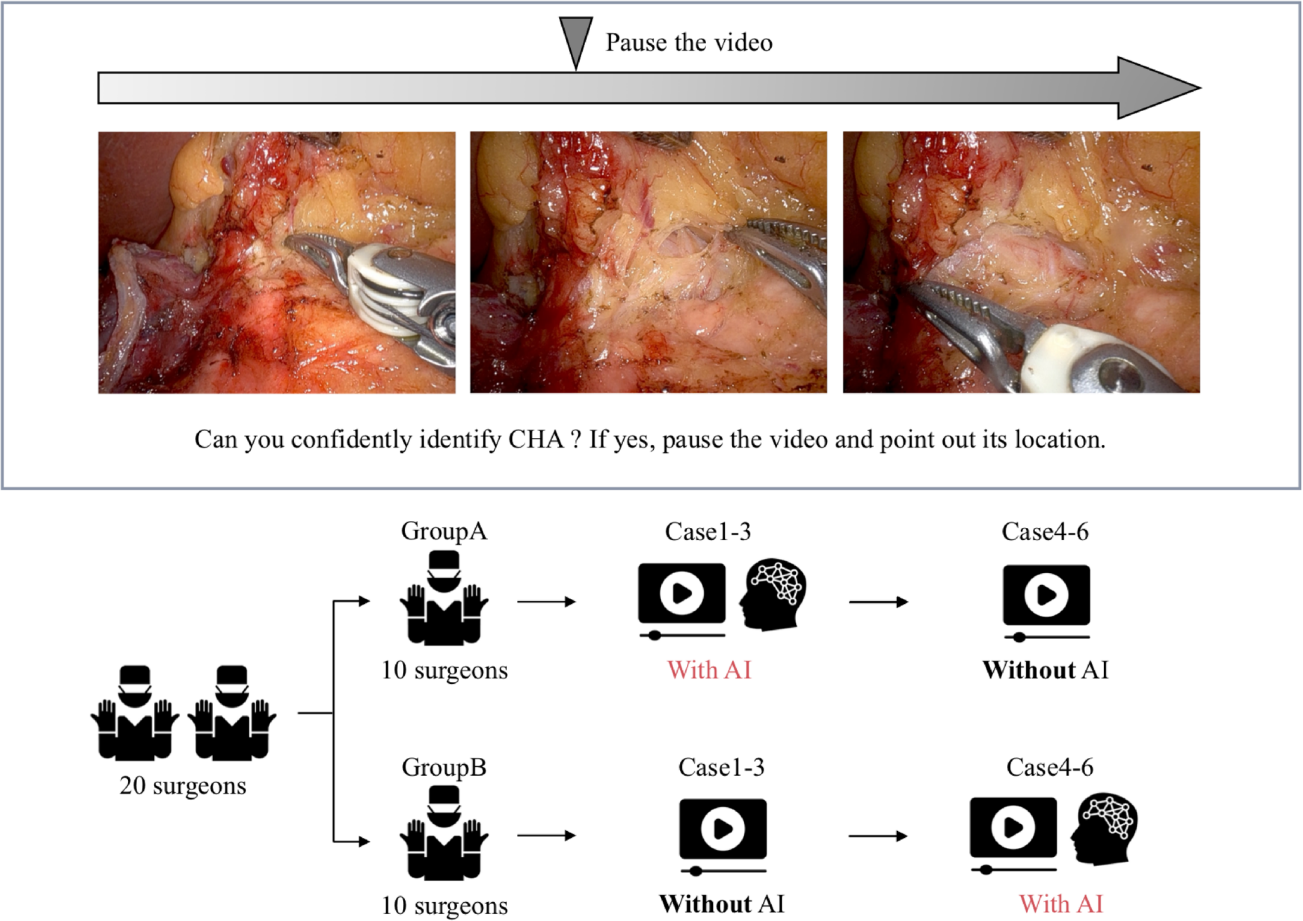
Workflow of experiment 2 assessing the time to CHA identification with and without AI assistance.

To assess the usability of the AI system for surgeons in clinical practice, we set up the following two experiments to compare the outcomes between surgeons who used AI and those who did not (Figs. 1 and 2): experiment 1, which is the verification of peritoneal incision line selection, and experiment 2, which is the verification of the time for CHA recognition.

Twenty surgeons who had assisted in approximately 100 gastrectomies participated in both experiments; the median number performed as the primary operator was 3 (range, 0–13), and 75% were board-certified. The participant characteristics are presented in Table 1.

**Table 1:** Characteristics of the participating surgeons.

Characteristic	Overall (*n* = 20)	Group A (*n* = 10)	Group B (*n* = 10)
Postgraduate year, median (range)	6 (3–7)	6 (3–7)	6 (3–7)
Board-certified surgeon, n (%)	12 (75%)	6 (60%)	6 (60%)
Number of gastrectomies performed, median (range)	3.0 (0–13)	3 (0–10)	1.5 (0–13)
Previously exposed to AI-based surgical systems, n (%)	8 (40%)	3 (30%)	5 (50%)

AI, artificial intelligence

#### Experiment 1

Four surgical videos from two hospitals that were independent of the training dataset were used to assess AI utility. The expert surgeon extracted 10- to 15-second scenes and still images immediately before peritoneal incision in each case, creating four original videos and still images; we also created four corresponding AI-enhanced videos and still images. First, participants watched the original video and then drew the peritoneal incision line that they considered best on the still image. Next, they watched an AI-enhanced video of the same case and drew a line on the AI-enhanced still image. They performed this task in all four cases and were only allowed to watch each video once, without looking back. Three blinded expert surgeons who had no involvement in AI model development or data annotation independently assessed the incision lines on a 5-point scale (1 = clearly unsafe, 5 = ideal; Supplementary Table S1). To ensure the reliability of these subjective assessments, interrater reliability among the three expert surgeons was evaluated using weighted intraclass correlation coefficients (ICCs). The primary outcome was the proportion of scores of 1–2, and the secondary outcome was the mean expert score.

#### Experiment 2

Six surgical videos from two hospitals that were independent of the training dataset and experiment 1 were used to assess AI utility. An expert surgeon extracted scenes ranging from peritoneal incision to division of the LGA, creating six original videos; additionally, we created six corresponding AI-enhanced videos. Twenty participants were allocated to two groups using stratified randomization according to their years of surgical experience in a crossover design: group A reviewed videos of the first three cases (cases 1–3) without AI assistance, followed by those of the next three cases (cases 4–6) with AI assistance, and group B reviewed the first three cases with AI assistance and the subsequent three cases without AI assistance. The participants were instructed to stop viewing the videos when they were confident that the CHA had been identified, following a previously reported approach [22]. The primary outcome was the time to CHA identification. In exploratory analyses, the case-specific effects of AI assistance were examined to assess consistency across cases.

###  Statistical analyses

Categorical data were analyzed using mixed-effects logistic regression models, whereas ordinal data were analyzed using mixed-effects ordinal logistic regression models. Continuous variables were analyzed using linear mixed-effects models (LMMs) or the Mann–Whitney U test, as appropriate. Learning effects in Experiment 2 were evaluated using an LMM with case number as a fixed effect. All statistical analyses were performed using R version 4.3.1 (R Foundation for Statistical Computing, Vienna, Austria), with a two-sided *P*-value of < 0.05 considered statistically significant.

## Results

### AI model performance for anatomical recognition

**Fig. 3:**
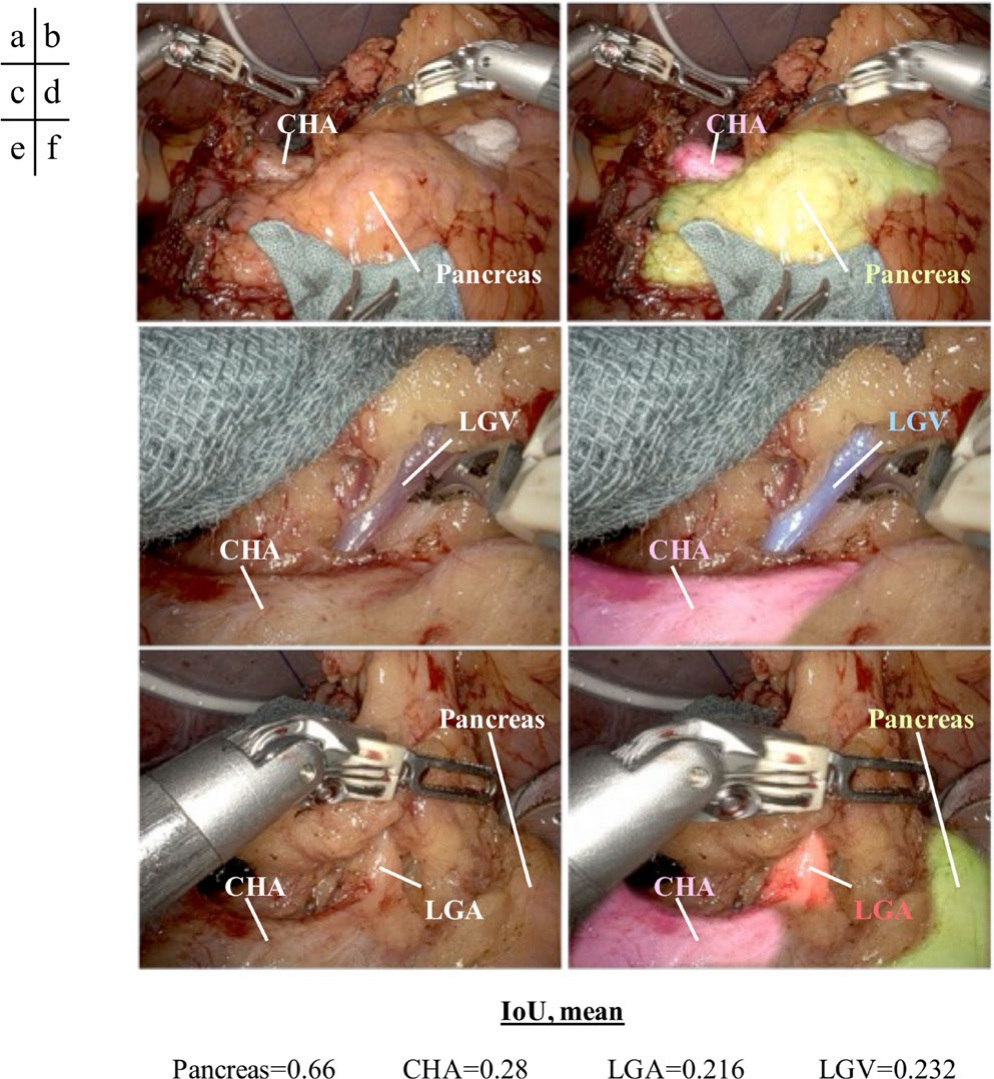
Intraoperative images without and with AI assistance and IoU values for the AI model.

During suprapancreatic lymph node dissection, the IoUs for AI recognition of the pancreas, CHA, LGA, and LGV were 0.66, 0.28, 0.216, and 0.232, respectively (Fig. 3). To visualize the performance of the AI model, representative videos of AI recognition are shown in Supplementary Video).

### Surgeons’ performance during anatomical recognition with or without AI

#### Experiment 1

**Fig. 4:**
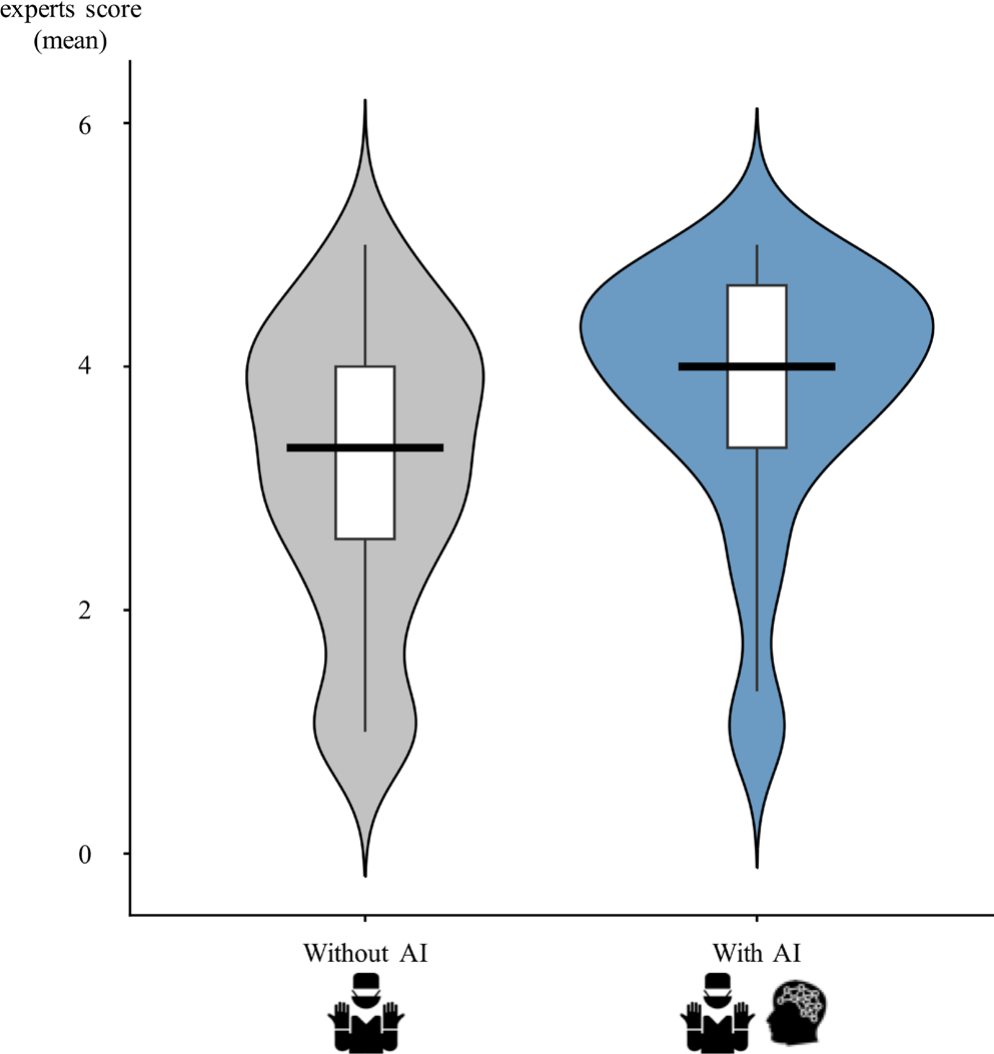
Violin plot of mean expert scores for peritoneal incision line selection with and without AI assistance. Scores range from 1 (clearly unsafe) to 5 (ideal).

The interrater reliability among the three expert surgeons was high, with weighted ICCs of 0.639 for single measures (ICC [2,1]) and 0.841 for average measures (ICC [2,k]). After adjusting for inter-surgeon and inter-case variabilities, the proportion of the incision lines rated as scores 1–2, which show unsafe lines, was significantly lower in the AI-assisted condition than in the non-assisted condition (odds ratio, 0.25; 95% confidence interval [CI], 0.15–0.41; *P* < 0.001). Similarly, after adjustment, the AI-assisted condition was associated with a significantly higher mean expert score than the non-assisted condition (estimated difference, 0.64 points; 95% CI, 0.39–0.89; *P* < 0.001). The distribution of the mean expert scores is shown in Fig. 4.

#### Experiment 2

**Fig. 5:**
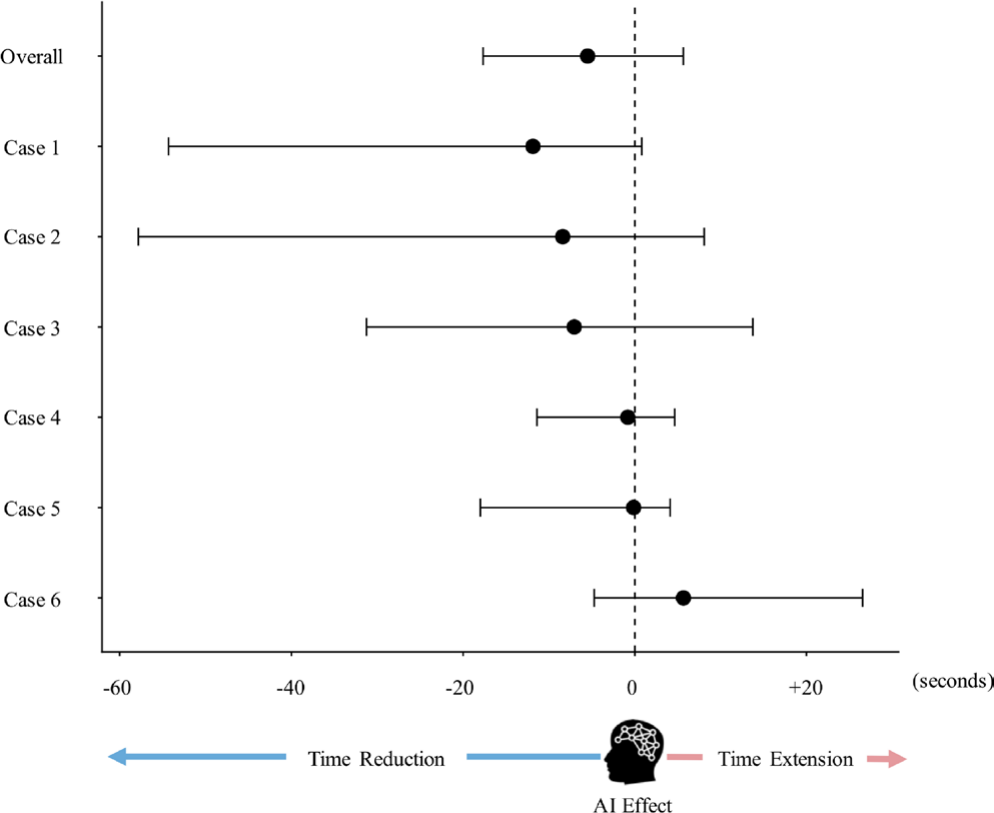
Case-specific differences in median time to CHA identification with 95% confidence intervals. The differences were calculated as AI-assisted minus non-assisted conditions.

After adjusting for inter-surgeon and inter-case variabilities, AI assistance was associated with a significant reduction in the time to CHA identification compared with the non-assisted condition (estimated difference, −9.5 s; 95% CI, −16.81 to −2.20; *P* = 0.0124). No significant practice effect was identified (β = −0.95 s/case; 95% CI, − 3.46 to 1.57; *P* = 0.460). CHA was correctly identified in all trials under both conditions. The case-specific median times for CHA identification are summarized in Fig. 5 and Supplementary Table S2. In five of the six cases, the AI-assisted condition resulted in shorter identification times, whereas in one case, the non-assisted condition resulted in shorter identification times. No statistically significant differences were observed in case-specific comparisons.

## Discussion

In this study, we established an AI-based anatomical recognition system for suprapancreatic lymph node dissection during RDG. This study demonstrated that our AI system supported the surgeons’ intraoperative decision-making in determining the peritoneal incision line, resulting in a significant reduction in unsafe incision lines. Moreover, AI assistance dramatically shortened the time required to identify the CHA. To the best of our knowledge, this is the first IDEAL Stage 0 study suggesting that an AI system can support surgeons’ intraoperative decision-making during technically demanding phases of surgery.

Various AI systems have been developed to support the surgeons’ anatomical recognition across different surgical procedures, including laparoscopic cholecystectomy, robotic esophagectomy, robotic pancreatoduodenectomy, and RDG [18, 23–25]. Many systems have demonstrated educational utility, particularly for medical students [20, 26]. Okumura et al. reported that an AI system improved medical students’ recognition of the pancreas and selection of dissection lines during RDG [19]. However, these studies have limitations, as their utility to surgeons has not yet been investigated. However, in our previous study, we showed that AI assistance improved the accuracy of surgeons in recognizing organ boundaries during RDG [21]. From the perspective of the IDEAL framework, the impact of improved anatomical recognition on intraoperative decision-making has remained unclear [27, 28].

Our AI system supported the surgeons’ intraoperative decision-making in determining the peritoneal incision line, resulting in a significant reduction in unsafe incision lines and an overall improvement in incision line quality. Determining the peritoneal incision line represents a critical intraoperative judgment because inappropriate incision lines may directly expose the pancreas to mechanical or thermal injuries. Therefore, a reduction in unsafe incision lines suggests a potential decrease in pancreatic injury, thereby mitigating the risk of postoperative pancreatic fistula. Previous studies have shown that cognitive burden impairs clinical decision-making and surgical performance [29]. By providing anatomical information, our system may reduce the cognitive burden required for continuous anatomical inference. When AI accuracy is limited, surgeons must allocate additional cognitive resources to verify AI outputs before acting upon them [30]. Further studies are needed to evaluate the cognitive impact of AI assistance in surgery.

Furthermore, our system dramatically shortened the time required by surgeons to identify the CHA. The early identification of critical anatomical structures may reduce operative blood loss by decreasing the mobilization and dissection of fatty tissues. Although we previously reported that the AI system reduces the time required to identify the recurrent laryngeal nerve during esophagectomy, evidence of similar early identification effects in abdominal surgery has been limited [31]. Therefore, the present findings support the potential of AI to facilitate the earlier identification of critical structures in abdominal procedures.

In this study, AI assistance improved surgeons’ intraoperative decision-making for pancreas and CHA recognition, despite the difference in IoU values between these structures. The IoU is widely used to evaluate anatomical recognition AI; however, small structures such as vessels and nerves tend to yield lower values because of their limited number of target pixels [23, 32, 33]. Favorable surgeon assessments, even with low IoU values, have also been reported for the pelvic autonomic nerve (IoU, 0.29) and the pancreas (IoU, 0.46), consistent with our results [16, 26]. As no clinical IoU cutoff has been established for surgical AI, further work is needed to develop surgical AI–specific evaluation metrics.

This study has several limitations. First, this study used recorded surgical videos of RDG and was not conducted in actual clinical practice. Our system has been confirmed to process surgical images in real-time in a research setting; however, clinical implementation will require regulatory approval as a medical device, as well as prospective evaluation of its effects on clinical outcomes such as pancreatic fistula, blood loss, operative time, and complications. Second, in experiment 1, the assessment was based on the subjective judgment of expert surgeons. Although good interrater reliability was observed, residual subjectivity could not be entirely excluded. Third, in experiment 2, the surgeons were allocated to the two study sequences in a balanced but not fully randomized manner, which may have introduced selection bias, and CHA identification time may have been affected by individual differences in video-pausing behavior. Finally, external validation with independent datasets from other institutions is needed to confirm the generalizability of the AI model.

In conclusion, this study provides preliminary evidence that AI-based anatomical recognition may support surgeons’ intraoperative decision-making during suprapancreatic lymph node dissection. Improved anatomical recognition may contribute to safer surgical performance and earlier identification of critical anatomical structures. These findings suggest that this system may be useful as a clinical decision-support system for surgeons. Further prospective studies are warranted to clarify its impact on surgical outcomes and routine clinical practice.

## Supplementary Information

Below is the link to the electronic supplementary material.

Supplementary Video.

Supplementary Tables.

## Data Availability

The data that support the findings of this study are available from the corresponding author upon reasonable request.

## References

[1] Bray F, Laversanne M, Sung H, Ferlay J, Siegel RL, Soerjomataram I, et al. Global cancer statistics 2022: GLOBOCAN estimates of incidence and mortality worldwide for 36 cancers in 185 countries. CA Cancer J Clin. 2024;74:229–263. 38572751 10.3322/caac.21834

[2] Sasako M. Progress in the treatment of gastric cancer in Japan over the last 50 years. Ann Gastroenterol Surg. 2020;4:21–9. 32021955 10.1002/ags3.12306 PMC6992673

[3] Japanese Gastric Cancer Association. Japanese Gastric Cancer Treatment Guidelines 2021 (6th edition). Gastric Cancer. 2023;26(1):1–25. 36342574 10.1007/s10120-022-01331-8 PMC9813208

[4] Shibasaki S, Suda K, Hisamori S, Obama K, Terashima M, Uyama I. Robotic gastrectomy for gastric cancer: systematic review and future directions. Gastric Cancer. 2023;26:325–38. 37010634 10.1007/s10120-023-01389-y

[5] Parisi A, Reim D, Borghi F, Nguyen NT, Qi F, Coratti A, et al. Minimally invasive surgery for gastric cancer: a comparison between robotic, laparoscopic and open surgery. World J Gastroenterol. 2017;23:2376–84. 28428717 10.3748/wjg.v23.i13.2376 PMC5385404

[6] Ryan S, Tameron A, Murphy A, Hussain L, Dunki-Jacobs E, Lee DY. Robotic versus laparoscopic gastrectomy for gastric adenocarcinoma: propensity-matched analysis. Surg Innov. 2020;27(1):26–31. 31441711 10.1177/1553350619868113

[7] Li ZY, Zhou YB, Li TY, Li JP, Zhou ZW, She JJ, et al. Robotic gastrectomy versus laparoscopic gastrectomy for gastric cancer: a multicenter cohort study of 5402 patients in China. Ann Surg. 2023;277:e87–95. 34225299 10.1097/SLA.0000000000005046

[8] Suda K, Yamamoto H, Nishigori T, Obama K, Yoda Y, Hikage M, et al. Safe implementation of robotic gastrectomy for gastric cancer under the requirements for universal health insurance coverage: a retrospective cohort study using a nationwide registry database in Japan. Gastric Cancer. 2022;25:438–49. 34637042 10.1007/s10120-021-01257-7 PMC8505217

[9] Lu J, Zheng CH, Xu BB, Xie JW, Wang JB, Lin JX, et al. Assessment of robotic versus laparoscopic distal gastrectomy for gastric cancer: a randomized controlled trial. Ann Surg. 2021;273:858–67. 32889876 10.1097/SLA.0000000000004466

[10] Shimoike N, Nishigori T, Yamashita Y, Kondo M, Manaka D, Kadokawa Y, et al. Safety assessment of robotic gastrectomy and analysis of surgical learning process: a multicenter cohort study. Gastric Cancer. 2022;25:817–26. 35416523 10.1007/s10120-022-01289-7

[11] Tokunaga M, Kurokawa Y, Machida R, Sato Y, Takiguchi S, Doki Y, et al. Impact of postoperative complications on survival outcomes in patients with gastric cancer: exploratory analysis of a randomized controlled JCOG1001 trial. Gastric Cancer. 2021;24:214–23. 32601909 10.1007/s10120-020-01102-3

[12] Dindo D, Demartines N, Clavien PA. Classification of surgical complications: a new proposal with evaluation in a cohort of 6336 patients and results of a survey. Ann Surg. 2004;240:205–13. 15273542 10.1097/01.sla.0000133083.54934.ae PMC1360123

[13] Clavien PA, Barkun J, de Oliveira ML, Vauthey JN, Dindo D, Schulick RD, et al. The Clavien-Dindo classification of surgical complications: 5 year experience. Ann Surg. 2009;250:187–96. 19638912 10.1097/SLA.0b013e3181b13ca2

[14] Suliburk JW, Buck QM, Pirko CJ, Massarweh NN, Barshes NR, Singh H, et al. Analysis of human performance deficiencies associated with surgical adverse events. JAMA Netw Open. 2019;2:e198067. 31365107 10.1001/jamanetworkopen.2019.8067 PMC6669897

[15] Khan DZ, Valetopoulou A, Das A, Hanrahan JG, Williams SC, Bano S, et al. Artificial intelligence assisted operative anatomy recognition in endoscopic pituitary surgery. NPJ Digit Med. 2024;7(1):314. 39521895 10.1038/s41746-024-01273-8 PMC11550325

[16] Nakamura T, Kobayashi N, Kumazu Y, Fukata K, Murakami M, Kohno S, et al. Precise highlighting of the pancreas by semantic segmentation during robot-assisted gastrectomy: visual assistance with artificial intelligence for surgeons. Gastric Cancer. 2024;27:869–875. 38573374 10.1007/s10120-024-01495-5

[17] Kumazu Y, Kobayashi N, Senya S, Negishi Y, Kinoshita K, Fukui Y, et al. AI-based visualization of loose connective tissue as a dissectable layer in gastrointestinal surgery. Sci Rep. 2025;15:152. 39747477 10.1038/s41598-024-84044-5 PMC11695969

[18] Aoyama Y, Matsunobu Y, Etoh T, Suzuki K, Fujita S, Aiba T, et al. Artificial intelligence for surgical safety during laparoscopic gastrectomy for gastric cancer: indication of anatomical landmarks related to postoperative pancreatic fistula using deep learning. Surg Endosc. 2024;38:5601–12. 39093411 10.1007/s00464-024-11117-x

[19] Okumura S, Tsunoda S, Hisamori S, Kitano S, Ueno K, Sakaguchi M, et al. Artificial intelligence-supported system of surgical anatomy recognition may facilitate the understanding of gastrointestinal surgery for medical students. Surg Endosc. 2025;39:7078–7086. 40944744 10.1007/s00464-025-12205-2 PMC12500820

[20] Nakao E, Igeta M, Kobayashi N, Kumazu Y, Otani Y, Murakami M, et al. Effectiveness of artificial intelligence-based visualization for surgical anatomy education: a cluster quasirandomized controlled trial. Surgery. 2025;188:109723. 41005002 10.1016/j.surg.2025.109723

[21] Ikeda J, Takeuchi M, Kawakubo H, Yura M, Kinoshita T, Morito A, et al. Assessment of AI-driven anatomical recognition in robotic gastrectomy: a multicenter retrospective analysis. Surg Endosc. 2025;39(12):8349–8356. 41003776 10.1007/s00464-025-12248-5

[22] Mizota T, Anton NE, Stefanidis D. Surgeons see anatomical structures faster and more accurately compared to novices: development of a pattern recognition skill assessment platform. Am J Surg. 2019;217:222–227. 30482478 10.1016/j.amjsurg.2018.10.011

[23] Madani A, Namazi B, Altieri MS, Hashimoto DA, Rivera AM, Pucher PH, et al. Artificial intelligence for intraoperative guidance: using semantic segmentation to identify surgical anatomy during laparoscopic cholecystectomy. Ann Surg. 2022;276:363–369. 33196488 10.1097/SLA.0000000000004594 PMC8186165

[24] Furube T, Takeuchi M, Kawakubo H, Noma K, Maeda N, Daiko H, et al. Usefulness of an artificial intelligence model in recognizing recurrent laryngeal nerves during robot-assisted minimally invasive esophagectomy. Ann Surg Oncol. 2024;31:9344–9351. 39266790 10.1245/s10434-024-16157-0

[25] Tomita K, Takeuchi M, Takayama M, Kim MP, Maxwell JE, Snyder RA, et al. Development of a novel artificial intelligence model to recognize vascular anatomy during robotic pancreatoduodenectomy. Br J Surg. 2025;112(11):znaf255. 41236618 10.1093/bjs/znaf255

[26] Kinoshita K, Maruyama T, Kobayashi N, Imanishi S, Maruyama M, Ohira G, et al. An artificial intelligence-based nerve recognition model is useful as surgical support technology and as an educational tool in laparoscopic and robot-assisted rectal cancer surgery. Surg Endosc. 2024;38:5394–5404. 39073558 10.1007/s00464-024-10939-z PMC11362368

[27] McCulloch P, Altman DG, Campbell WB, Flum DR, Glasziou P, Marshall JC, et al. No surgical innovation without evaluation: the IDEAL recommendations. Lancet. 2009;374:1105–1112. 19782876 10.1016/S0140-6736(09)61116-8

[28] Barkun JS, Aronson JK, Feldman LS, Maddern GJ, Strasberg SM, Altman DG, et al. Evaluation and stages of surgical innovations. Lancet. 2009;374:1089–1096. 19782874 10.1016/S0140-6736(09)61083-7

[29] Almukhtar A, Caddick V, Naik R, Goble M, Mylonas G, Darzi A, et al. Objective assessment of cognitive workload in surgery: a systematic review. Ann Surg. 2025;281:942–951. 38847099 10.1097/SLA.0000000000006370 PMC12061381

[30] Yu F, Moehring A, Banerjee O, Salz T, Agarwal N, Rajpurkar P. Heterogeneity and predictors of the effects of AI assistance on radiologists. Nat Med. 2024;30:837–49. 38504016 10.1038/s41591-024-02850-w PMC10957478

[31] Furube T, Takeuchi M, Kawakubo H, Noma K, Maeda N, Daiko H, et al. Impact of artificial intelligence on the timing of recurrent laryngeal nerve recognition during robot-assisted minimally invasive esophagectomy. Ann Surg Oncol. 2025;32:6366–6373. 40569356 10.1245/s10434-025-17649-3

[32] Maier-Hein L, Reinke A, Godau P, Tizabi MD, Buettner F, Christodoulou E, et al. Metrics reloaded: recommendations for image analysis validation. Nat Methods. 2024;21:195–212. 38347141 10.1038/s41592-023-02151-z PMC11182665

[33] Kolbinger FR, Rinner FM, Jenke AC, Carstens M, Krell S, Leger S, et al. Anatomy segmentation in laparoscopic surgery: comparison of machine learning and human expertise—an experimental study. Int J Surg. 2023;109:2962–74. 37526099 10.1097/JS9.0000000000000595 PMC10583931

