# Supplementary material for: Artificial intelligence–based anatomical recognition improves surgeon decision-making during robotic gastrectomy
Source: Gastric Cancer. 2026 May 19;29(4):874–81. doi: 10.1007/s10120-026-01756-5 (PMC13315160; doi:10.1007/s10120-026-01756-5)
Supplement: Supplementary file 2 — Supplementary Tables. [file 10120_2026_1756_MOESM2_ESM.docx]

**Supplementary Table S1**

Scoring criteria used for expert evaluation of experiment 1.

| Score | Category | Description |
| --- | --- | --- |
| 5 | Ideal | No modification required; optimal safety and adequacy. |
| 4 | Acceptable | No modification required; acceptable for proceeding, though not optimal. |
| 3 | Modification required | Modification of the incision line is required to safely and  adequately perform lymph node dissection. |
| 2 | Potentially unsafe | Proceeding carries a clear risk of injury; major modification is required  before proceeding. |
| 1 | Clearly unsafe | Clearly unsafe to proceed; imminent risk of injury to critical structures. |

**Supplementary Table S2**

Case-specific median time to common hepatic artery identification with and without AI assistance and the corresponding differences.

AI, artificial intelligence.

| Case | With AI (seconds)  median (i.q.r.) | Without AI (seconds)  median (i.q.r.) | Differene (With AI– Without AI) (seconds)  median (95% c.i.) |
| --- | --- | --- | --- |
| Case 1 | 35.09 [34.53 - 36.06] | 46.96 [34.87 - 85.95] | -11.87 (-54.30 - 0.76) |
| Case 2 | 50.75 [49.25 - 59.02] | 59.14 [56.42 - 78.51] | -8.40 (-57.85 - 7.69) |
| Case 3 | 67.36 [50.71 - 78.11] | 74.42 [62.57 - 82.13] | -7.06 (-30.78 - 14.18) |
| Case 4 | 30.56 [29.72 - 34.09] | 31.38 [28.06 - 39.72] | -0.82 (-11.79 - 4.88) |
| Case 5 | 43.89 [43.23 - 44.45] | 44.03 [40.56 - 56.25] | -0.14 (-18.01 - 4.07) |
| Case 6 | 63.08 [60.39 - 66.49] | 57.41 [38.91 - 65.06] | 5.67 (-4.97 - 26.84) |
| Overall | 44.12 [34.56 - 62.06] | 49.62 [35.83 - 72.64] | -5.50 (-17.79 - 5.87) |
